# Cardiovascular reserve as an integrative physiological construct: Current evidence, emerging technologies and future directions

**DOI:** 10.1111/cpf.70094

**Published:** 2026-10-04

**Authors:** Uri Gabbay, Shai Fein, Michael J. Drescher

**Affiliations:** ^1^ Gray Faculty of Medicine and Health Sciences Tel Aviv University Tel Aviv Israel; ^2^ Department of Anesthesiology Beilinson Medical Center Petach Tikva Israel; ^3^ Department of Emergency Medicine Beilinson Medical Center Petach Tikva Israel

**Keywords:** cardiovascular reserve, Cardiovascular Reserve Index (CVRI), compensatory reserve, Compensatory Reserve Index (CRI), Compensatory Reserve Metric (CRM), haemorrhage, physiological reserve, shock

## Abstract

Cardiovascular reserve represents the integrated physiological capacity of the cardiovascular system to maintain tissue perfusion during increasing physiological stress. Although reserve has long been recognized conceptually, its direct assessment remains challenging. No universally accepted definition or gold‐standard measure of cardiovascular reserve currently exists. Nevertheless, if cardiovascular reserve can be estimated reliably, reserve‐based monitoring could permit earlier recognition of impending circulatory deterioration before conventional physiological indicators become abnormal. Among the reserve‐monitoring technologies currently under investigation, the Compensatory Reserve Metric (CRM) and the Cardiovascular Reserve Index (CVRI) represent two distinct approaches to estimating remaining cardiovascular reserve. This review examines the physiological basis of reserve‐based monitoring, critically evaluates the available experimental and clinical evidence supporting CRM and CVRI, assesses their strengths and limitations and outlines priorities for future research and potential clinical applications. CRM has demonstrated strong performance in experimental haemorrhage models and remains the most extensively studied reserve‐monitoring technology in low output conditions. Although evidence supporting CVRI is more limited, findings from cardiopulmonary exercise testing, haemorrhage and heat stress suggest that it may reflect progressive depletion of cardiovascular reserve across diverse forms of circulatory and physiological stress. Although seemingly promising, current evidence remains limited by small clinical cohorts, heterogeneous study designs and limited independent validation.

## 1 INTRODUCTION

Cardiovascular reserve is a fundamental physiological property that determines the ability of the cardiovascular system to respond to increasing metabolic and circulatory demands. Although the concept underlies exercise physiology, heart failure, haemorrhage, aging and critical illness, direct assessment of cardiovascular reserve remains challenging. Recognition of impending circulatory collapse before overt deterioration remains a central challenge in acute medicine. Conventional physiological measurements, including heart rate, blood pressure, respiratory rate, oxygen saturation and cardiac output estimates, primarily reflect a patient's current physiological status rather than the remaining capacity to compensate for ongoing physiological stress (Cannon, 2018; Kaufmann et al., 2020; Vincent and De Backer, 2013). Compensatory mechanisms may maintain apparently normal physiological measurements despite substantial physiological stress, allowing patients to preserve acceptable hemodynamic parameters while progressively exhausting their physiological reserve. Consequently, hypotension and other overt manifestations of circulatory failure often occur relatively late during circulatory deterioration (Cannon, 2018; Kaufmann et al., 2020; Vincent and De Backer, 2013).

To improve early recognition of deterioration, several composite physiological indices and early warning systems have been developed, including the Shock Index, the Modified Early Warning Score (MEWS) and the National Early Warning Score (NEWS) (Cannon, 2018; Rady et al., 1992; Royal College of Physicians, 2017; Smith et al., 2013; Subbe et al., 2001). Although these approaches generally outperform individual vital signs, they detect deterioration only after much of the available compensatory reserve has already been depleted (Cannon, 2018; Rady et al., 1992; Royal College of Physicians, 2017; Smith et al., 2013; Subbe et al., 2001). Several physiological markers, including heart‐rate variability, perfusion index, pleth variability index and near‐infrared spectroscopy, have also been investigated as indicators of hemodynamic compromise (Cannesson et al., 2008; Cohn et al., 2007; Schlotman et al., 2020).

These limitations have stimulated growing interest in estimating physiological reserve as a complementary framework for understanding impending deterioration. Rather than focusing exclusively on current physiological performance, reserve‐based concepts seek to estimate the remaining capacity of integrated cardiovascular compensatory mechanisms to maintain homoeostasis under increasing physiological stress. Cardiovascular reserve has long been recognized in cardiovascular physiology, particularly in exercise physiology and heart failure. Conceptually, it reflects the balance between maximal recruitable cardiovascular capacity and concurrent physiological demand. Reserve therefore declines either when cardiovascular capacity is reduced (e.g., haemorrhage or heart failure) or when physiological demand increases (e.g., exercise, heat stress or sepsis). Dynamic compensatory mechanisms, including the Frank‐Starling mechanism, sympathetic activation, peripheral vasoconstriction and neurohormonal responses, maintain tissue perfusion during increasing physiological stress, illustrating the integrated nature of cardiovascular compensation (Hartupee and Mann, 2017; Sterling and Eyer, 1988; Triposkiadis et al., 2009). Building on this physiological foundation, the cardiovascular reserve hypothesis proposed that progressive depletion of physiological reserve precedes overt physiological instability and provides a conceptual framework for understanding exercise capacity, heart failure and shock (Gabbay et al., 2014).

These limitations have stimulated interest in reserve‐based monitoring. The conceptual distinction between conventional physiological monitoring and reserve‐based monitoring is illustrated in Figure 1.

**Figure 1:**
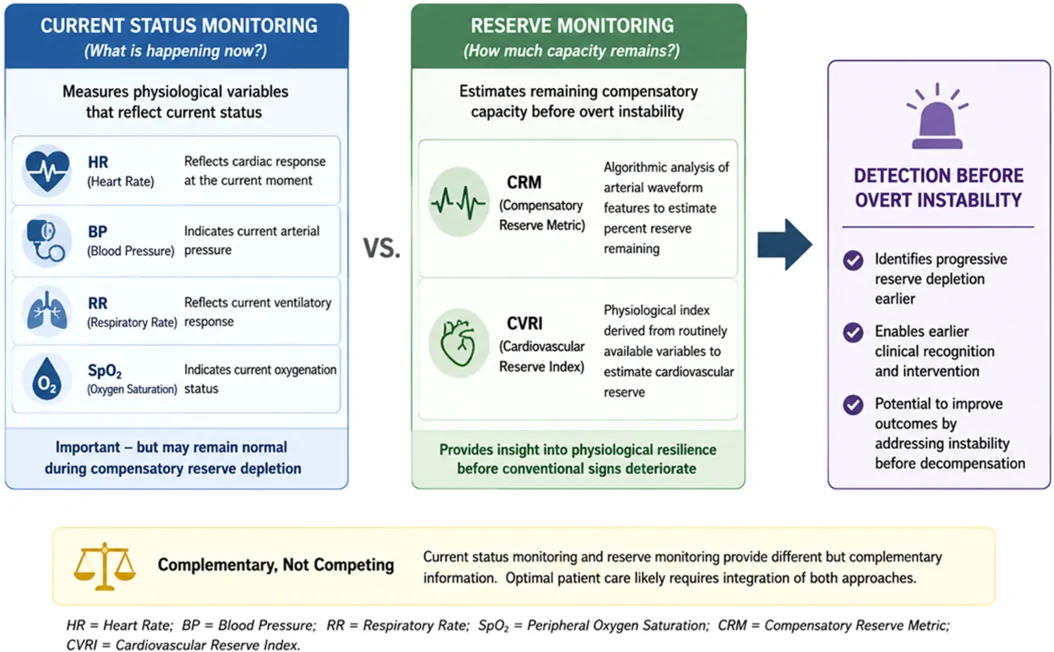
Conceptual comparison of conventional physiological monitoring and reserve‐based monitoring. Conventional monitoring evaluates current physiological status, whereas reserve‐based monitoring estimates remaining cardiovascular reserve. This conceptual difference may allow for earlier recognition of impending circulatory deterioration before conventional physiological variables become abnormal.

Among the reserve‐monitoring technologies described to date, the Compensatory Reserve Metric (CRM) and the Cardiovascular Reserve Index (CVRI) represent the two principal approaches currently under investigation. Although they employ different physiological models and analytical methods, both aim to estimate remaining cardiovascular reserve before overt circulatory deterioration develops.

This narrative review summarizes the physiological foundations of reserve‐based monitoring, critically reviews the available evidence for CRM and CVRI, compares their physiological concepts, clinical evidence, practical implementation and identifies priorities for future research and clinical translation.

## 2 METHODS

This review was conducted in accordance with accepted methodological approaches for narrative reviews that emphasize conceptual synthesis, critical interpretation and integration of emerging evidence rather than formal evidence aggregation (Baethge et al., 2019; Kelley and D'Souza, 2025). Because the terms Compensatory Reserve Metric (CRM) and Compensatory Reserve Index (CRI) have been used interchangeably by the same research group to describe closely related arterial waveform‐based reserve‐monitoring algorithms with highly correlated performance, literature searches for CRM also included the term ‘Compensatory Reserve Index’.

A structured literature search was performed in PubMed and Google Scholar through May 2026 using combinations of the following predefined keywords: ‘cardiovascular reserve’, ‘circulatory reserve’, ‘cardiovascular reserve index’, ‘compensatory reserve metric’, ‘compensatory reserve index’, ‘physiological reserve’, ‘circulatory deterioration’, ‘physiological deterioration’, ‘haemorrhage’ and ‘shock’. Reference lists of relevant publications were also screened to identify additional eligible studies. Only English‐language publications were included.

The objective of this review was to synthesize physiological rationale, methodological development and emerging clinical evidence for reserve‐based monitoring rather than to perform quantitative evidence synthesis; therefore, formal systematic‐review methodology was not applied. Studies were selected based on their relevance to physiological principles, methodological development, validation or clinical application of reserve‐based monitoring. Because relatively few original investigations have evaluated reserve‐monitoring technologies, all identified English‐language publications and conference abstracts that were directly relevant to CRM/CRI, CVRI or related reserve‐monitoring concepts were considered and critically appraised.

Studies were excluded when they did not address cardiovascular or compensatory reserve, reserve estimation or its physiological basis, or when they did not provide relevant methodological, physiological or clinical information.

### 2.1 Reserve‐based monitoring: Conceptual framework

Reserve‐based monitoring seeks to estimate remaining compensatory capacity rather than current physiological status. The reserve‐based monitoring paradigm is founded on the premise that circulatory deterioration reflects progressive depletion of compensatory reserve, providing a unifying physiological model for understanding exercise capacity, heart failure and shock across diverse physiological stressors (Gabbay et al., 2014). According to this framework, compensatory reserve declines progressively before hypotension, organ dysfunction or other overt manifestations of circulatory failure become clinically apparent (Gabbay et al., 2014).

Within this framework, physiological reserve is viewed as a continuum extending from optimal physiological health to overt circulatory failure. Healthy individuals possess substantial reserve and can tolerate considerable physiological stress, far above daily activities, before approaching compensatory limits. In contrast, chronic cardiovascular, pulmonary or systemic disease progressively narrows this reserve, reducing tolerance to additional physiological stress and, in advanced stages, even limiting the ability to perform ordinary daily activities, as exemplified by advanced heart failure. As compensatory mechanisms become progressively exhausted, the cardiovascular system eventually loses its ability to maintain adequate tissue perfusion, resulting in hemodynamic instability, organ dysfunction and ultimately shock. Thus, shock may represent the clinical manifestation of severe or exhausted compensatory reserve, at which compensatory mechanisms can no longer sustain adequate tissue perfusion rather than an abrupt physiological event.

This conceptual model provides the physiological rationale for reserve‐based monitoring. Because compensatory reserve may decline while conventional physiological measurements remain relatively preserved, estimating remaining reserve may allow earlier recognition of impending circulatory deterioration than conventional physiological monitoring alone.

### 2.2 Biological basis of cardiovascular reserve

Cardiovascular reserve reflects the integrated capacity of multiple cardiovascular and neurohumoral mechanisms to preserve arterial pressure, tissue perfusion and oxygen delivery during physiological stress. Rather than representing the reserve of a single organ, cardiovascular reserve emerges from the coordinated interaction of cardiac performance, vascular function, autonomic regulation, redistribution of blood flow and tissue oxygen utilization. Consequently, reserve is best viewed as an integrative physiological property of the entire cardiovascular system rather than as an isolated measure of cardiac function.

Acute circulatory stress activates integrated autonomic compensatory mechanisms, in which the arterial baroreflex plays a central role. Reduced baroreceptor firing during hypovolemia or impaired arterial filling increases sympathetic activity, producing tachycardia, enhanced myocardial contractility and peripheral vasoconstriction. These responses help maintain cardiac output and arterial pressure despite reductions in effective circulating volume.

As physiological stress increases, cardiovascular compensation becomes progressively more demanding. Initially, cardiac output is supported through increases in heart rate, preserved venous return and maintenance of stroke volume. Peripheral vasoconstriction increases systemic vascular resistance, thereby preserving arterial pressure even as cardiac output begins to decline. Consequently, hypotension is often a relatively late manifestation of progressive haemorrhage or other forms of circulatory stress.

The effectiveness of these compensatory responses depends on coordinated interactions between cardiac performance and vascular function. Adequate cardiovascular reserve therefore requires both preserved myocardial contractile capacity and appropriate vascular responsiveness to maintain effective tissue perfusion.

Neurohormonal activation, including the renin–angiotensin–aldosterone system and vasopressin release, contributes to longer‐term maintenance of vascular tone and intravascular volume but plays a less prominent role in the immediate circulatory augmentation that characterizes acute compensatory responses (Hartupee and Mann, 2017; Sterling and Eyer, 1988; Triposkiadis et al., 2009).

Ultimately, cardiovascular compensation serves to preserve tissue oxygen delivery in the face of increasing physiological demand. Although arterial pressure and cardiac output remain important determinants of circulatory performance, maintenance of adequate oxygen delivery depends on the integrated interaction of cardiac output, vascular resistance, blood volume, pulmonary oxygenation and regional blood‐flow distribution. Progressive exhaustion of reserve therefore reflects diminishing capacity to maintain adequate oxygen delivery rather than failure of any single physiological variable.

This integrated physiological behaviour explains why conventional hemodynamic variables may remain within normal limits despite substantial depletion of cardiovascular reserve. Cardiovascular reserve may decline either because cardiovascular capacity is reduced (e.g., haemorrhage or cardiac dysfunction) or because physiological demand increases (e.g., exercise, heat stress or infection). This framework explains why cardiovascular reserve is recruited across diverse physiological and pathological conditions. During exercise, reserve is progressively recruited to support increasing metabolic demand while remaining physiologically appropriate. In contrast, haemorrhage, sepsis, heart failure and severe heat stress progressively consume reserve because compensatory mechanisms must offset pathological disturbances in cardiovascular homoeostasis. Despite these differing mechanisms, reserve depletion reflects the common physiological challenge of maintaining adequate tissue perfusion under increasing stress.

Because cardiovascular compensation reflects an integrated physiological network rather than isolated mechanisms, individual physiological variables provide only partial information about remaining reserve. This provides the physiological rationale for reserve‐monitoring approaches that integrate multiple physiological signals rather than relying on any single hemodynamic variable. Once compensatory reserve is exhausted, cardiovascular compensation fails, resulting in hemodynamic instability, organ dysfunction and ultimately shock.

The concept of cardiovascular reserve also provides a physiological bridge between normal adaptation and disease. Healthy individuals possess substantial cardiovascular reserve that permits large increases in cardiovascular performance during exercise and other physiological stressors, allowing physiological demands to be met until maximal exercise capacity is reached. Aging, chronic cardiovascular disease, pulmonary disease and systemic illness progressively reduce this reserve, narrowing the margin between resting function and physiological failure. From this perspective, cardiovascular reserve represents the functional capacity of the integrated cardiovascular system to accommodate changing physiological demands while maintaining tissue perfusion and oxygen delivery. Progressive loss of reserve therefore provides a common physiological framework linking normal adaptation, aging, chronic disease and acute circulatory failure.

### 2.3 Reserve‐monitoring technologies

#### 2.3.1 Compensatory Reserve Metric (CRM)

The CRM was developed using lower‐body negative pressure (LBNP) models that simulate progressive central hypovolemia and haemorrhage. Machine‐learning algorithms analyse features of arterial pressure or photoplethysmographic waveforms to estimate remaining compensatory reserve, providing a continuously updated estimate of remaining cardiovascular reserve. Declining CRM values indicate progressive depletion of cardiovascular reserve before conventional physiological variables become abnormal (Convertino et al., 2023; Roden et al., 2024; Schlotman et al., 2020). The terms CRM and CRI have been used interchangeably by the same research group to describe closely related machine‐learning approaches that estimate remaining compensatory reserve from arterial waveform analysis (Convertino et al., 2023; Roden et al., 2024; Schiller et al., 2018; Schlotman et al., 2020).

### 2.4 Cardiovascular Reserve Index (CVRI)

The CVRI is a calculated index designed to estimate remaining cardiovascular reserve by integrating routinely available physiological variables, including mean arterial pressure, heart rate, respiratory rate and body surface area, into a single measure of compensatory capacity (Gabbay et al., 2015).

CVRI originated from a systems‐control model of cardiovascular regulation in which cardiovascular reserve was conceptualized as the integrated capacity of cardiac performance (stroke volume), vascular compensation (systemic vascular resistance) and autonomic regulation (baroreceptor sensitivity) to maintain physiological stability during stress. The conceptual framework and derivation are summarized in Data S1.

Initial pilot proof‐of‐concept studies of CVRI demonstrated physiological behaviour consistent with the cardiovascular reserve hypothesis. Subsequent studies evaluated CVRI across exercise, heat stress, experimental haemorrhage and trauma (Epstein et al., 2015; Gabbay et al., 2019; Nadler et al., 2017; Schiffmann et al., 2021; Segel et al., 2017; Shaya et al., 2024).

### 2.5 Comparative evidence and conceptual relationship of CRM/CRI and CVRI

The individual experimental, preclinical and clinical studies evaluating CRM/CRI and CVRI are summarized in Table 1.

**Table 1:** Experimental and clinical evidence evaluating CRM/CRI and CVRI.

Technology	Reference	Study type	Model/population	Physiological stress/setting	Principal outcome	Main finding	Comparator	Evidence interpretation
**CRM/CRI**	Convertino et al., 2013	Experimental human	**201 healthy volunteers** undergoing progressive LBNP	Progressive central hypovolemia to hemodynamic decompensation	Prediction of individual progression to cardiovascular instability	CRI derived from arterial waveforms differentiated individual tolerance to central volume loss and identified progression toward decompensation before conventional changes in stroke volume or standard vital signs	Stroke volume and standard vital signs	**Physiological evaluation** and proof‐of‐concept under controlled hypovolemia, but not clinical haemorrhage
**CRM/CRI**	Schlotman et al., 2020	Experimental human	**101 healthy volunteers**	Progressive LBNP to decompensation	Detection of reduced central blood volume and onset of decompensation	CRM ROC AUC **0.93**, with sensitivity and specificity of **0.84/0.84** at the optimal threshold; performance exceeded HRV and heart‐rate‐complexity measures	HRV and HRC	**Experimental evaluation.** Supports CRM as an integrated marker of compensatory reserve
**CRM/CRI**	Hinojosa‐Laborde et al., 2016	Preclinical	**13 baboons**	Controlled haemorrhage (6.25%, 12.5%, 18.75%, 25% blood‐volume loss) and matched LBNP	Prediction of cardiovascular decompensation	CRI declined progressively during both haemorrhage and LBNP; haemorrhage and LBNP responses were highly correlated (***r*** = **0.96**); CRI showed greater specificity than stroke volume	Stroke volume; LBNP as experimental comparator	**Preclinical physiological evaluation.** Supports LBNP as a model of haemorrhage and demonstrates reserve tracking during actual blood loss
**CRM/CRI**	Convertino et al., 2023	Experimental human	**172 healthy volunteers**	Progressive LBNP to central hypovolemia/decompensation	Detection of reduced central blood volume and prediction of decompensation	CRM reached a clinically defined reserve threshold at a lower LBNP level than Shock Index; ROC AUC **0.95** for CRM versus **0.91** for Shock Index	Shock Index	**Experimental comparative evaluation.** Demonstrates earlier reserve signal than a conventional composite vital‐sign index
**CRM**	Convertino et al., 2022	Experimental human/algorithm comparison	**191 healthy volunteers**	Progressive LBNP simulating haemorrhage	Prediction of decompensated shock	CRM and CRI showed similar performance; ROC AUC **0.9268** for CRM and **0.9164** for CRI	CRI	**Methodological evaluation.** Supports robustness of arterial‐waveform reserve estimation across independently developed ML approaches
**CRM**	Roden et al., 2024	Experimental human/sensor validation	**49 healthy adults** (25 women)	Graded LBNP simulating haemorrhage	Evaluation of CRM obtained from pulse‐oximetry PPG	Pulse‐oximetry‐derived CRM tracked physiological changes and detected hemodynamic shock earlier than Shock Index, SBP and HR	PPG volume‐clamp CRM; SBP, HR, Shock Index	**Experimental/sensor evaluation.** Supports feasibility of a more portable noninvasive implementation
**CRM/CRI**	Johnson et al., 2018	Prospective clinical observational	**89 trauma patients** meeting trauma‐centre activation criteria at a single Level I trauma centre	Acute post‐traumatic haemorrhage risk	Haemorrhage‐related life‐saving intervention	CRI sensitivity **83%**, NPV **91%**; ROC AUC **0.83**, compared with **0.62** for SBP	SBP	**Preliminary clinical evaluation.** Encouraging performance in an activated trauma population, but single‐centre and relatively small
**CRM/CRI**	Latimer et al., 2023	Prospective prehospital observational	**696 adult injured patients** transported by EMS to a Level I trauma centre; **21%** met the study definition of haemorrhagic shock	Prehospital trauma/suspected haemorrhage	Haemorrhagic shock, defined by blood‐product administration prehospital or within 4 h of hospital arrival	Minimum CRI‐trend AUC 0.65; inferior to SBP (0.74) and Shock Index (0.72), although better than initial HR	SBP, HR, Shock Index	**Clinical evaluation with conflicting findings.** Demonstrates that promising experimental performance does not necessarily translate to superiority in heterogeneous prehospital trauma
**CVRI**	Gabbay et al., 2015	Retrospective clinical/physiological pilot	Three existing databases: **333 acute severe hospital admissions; 71 heart‐failure patients; 387 CPX participants**	Acute shock, heart failure and exercise‐capacity spectrum	Association between CVRI and severity of physiological/hemodynamic condition	CVRI was inversely associated with severity; lowest values occurred in shock on arrival and progressively higher values across less severe conditions	Clinical classification of shock, HF severity and exercise capacity	**Initial broad physiological evaluation.** Supports the proposed reserve construct across different physiological states, but retrospective and heterogeneous
**CVRI**	Segel et al., 2017	Retrospective physiological study	Patients undergoing CPET for diverse indications	Incremental exercise and recovery	CVRI trajectory and relationship to exercise capacity	Mean CVRI decreased from **0.81 at rest to 0.35 at peak exercise** and increased during recovery; pattern was consistent with the reserve hypothesis	Exercise stage/exercise capacity	**Physiological evaluation (proof‐of‐concept).** Demonstrates dynamic reserve utilization and recovery during exercise
**CVRI**	Nadler et al., 2017	Preclinical	Swine	Progressive controlled haemorrhage/exsanguination	Prediction of hemodynamic deterioration	CVRI declined progressively during blood loss and predicted hemodynamic deterioration before overt changes in conventional hemodynamic variables	Conventional hemodynamic measures	**Preclinical physiological evaluation.** Supports reserve tracking during actual blood loss, but animal model limits clinical inference
**CVRI**	Epstein et al., 2015	Clinical/experimental pilot abstract	Heat‐tolerance‐test participants	Exercise‐heat stress	Prediction of heat tolerance	Lower CVRI was associated with greater cardiovascular strain and heat intolerance	Heat‐tolerance classification	**Preliminary evaluation (proof‐of‐concept).** Conference‐abstract data; should be interpreted cautiously
**CVRI**	Schiffmann et al., 2021	Prospective clinical physiological study	**95 young men: 16 heat‐intolerant and 79 heat‐tolerant**	Standardized exercise‐heat stress/heat‐tolerance testing	Identification of heat intolerance	CVRI demonstrated greater reserve reduction in heat‐intolerant participants; partial ROC AUC in high‐sensitivity region **93.2%**	Dynamic heart‐rate‐reserve index	**Clinical physiological evaluation.** Extends CVRI evaluation beyond haemorrhage to increased physiological demand
**CVRI**	Gabbay et al., 2019	Historical prospective registry analysis	Israeli National Trauma Registry, **2015 trauma cohort**	Acute trauma	Early trauma death/survival to discharge	CVRI provided prognostic information beyond conventional trauma assessment and was evaluated against Shock Index	Shock Index/trauma variables	**Preliminary prognostic evaluation.** Registry‐based association; does not establish clinical utility or treatment benefit
**CVRI**	Shaya et al., 2024	Prospective observational clinical cohort	**71 adult trauma casualties** at a single Level I trauma centre; 5 massive haemorrhage on arrival, 5 developing haemorrhage, 61 without significant haemorrhage	Acute trauma and haemorrhage	Massive haemorrhage on arrival and subsequent developing haemorrhage	CVRI was associated with subsequent development of haemorrhage and demonstrated an AUC of 0.82; lactate AUC 0.88; most other variables lower AUC for developing haemorrhage	Lactate, Hb, vital signs, Shock Index	**Preliminary clinical evaluation.** Encouraging but small observational cohort; supports incremental prognostic information rather than clinical utility
**CVRI**	Raphaeli et al., 2022	Clinical conference abstract	**2122 adult ICU patients** hospitalized >48 h	Septic shock/critical illness	ICU mortality	Serial CVRI patterns were evaluated using machine‐learning models for mortality prediction	Admission characteristics/model‐based prediction	**Hypothesis‐generating only.** Conference abstract and not sufficient for definitive clinical validation

*Note*: Experimental studies primarily provide physiological or methodological validation and should not be interpreted as equivalent to clinical validation. Clinical observational and registry studies demonstrate diagnostic or prognostic associations but do not establish that reserve‐guided monitoring improves patient outcomes.

Abbreviations: AUC, area under the receiver‐operating‐characteristic curve; CPET/CPX, cardiopulmonary exercise testing; CRI, Compensatory Reserve Index; CRM, Compensatory Reserve Metric; CVRI, Cardiovascular Reserve Index; EMS, emergency medical services; Hb, haemoglobin; HR, heart rate; HRC, heart‐rate complexity; HRV, heart‐rate variability; ICU, intensive care unit; LBNP, lower‐body negative pressure; NPV, negative predictive value; PPG, photoplethysmography; ROC, receiver operating characteristic; SBP, systolic blood pressure.

Although CRM and CVRI were developed independently and employ fundamentally different analytical approaches, both were designed to estimate the same underlying physiological construct—remaining cardiovascular reserve. Whether these approaches quantify identical, complementary or partially distinct dimensions of cardiovascular reserve remains unknown and will require direct comparative investigation.

### 2.6 Experimental and clinical evaluation of CRM

The strongest evidence supporting the CRM derives from controlled LBNP, an established experimental model that simulates progressive central hypovolemia while permitting standardized investigation of cardiovascular compensatory responses (Convertino et al., 2023; Schlotman et al., 2020). Initial studies demonstrated that machine‐learning analysis of arterial waveforms could estimate progressive depletion of compensatory reserve before conventional physiological variables became abnormal (Convertino et al., 2023). These studies were conducted under controlled experimental conditions and therefore primarily provide physiological and methodological validation rather than clinical evaluation.

Subsequent investigations extended these findings beyond LBNP. In a controlled haemorrhage study involving 13 baboons, CRI declined progressively with blood loss and showed a strong correlation with the matched LBNP response (*r* = 0.96), supporting physiological correspondence between the experimental model and actual haemorrhage (Hinojosa‐Laborde et al., 2016). Additional controlled human LBNP studies in healthy volunteers demonstrated superior prediction of impending cardiovascular decompensation compared with lactate, muscle oxygen saturation, end‐tidal carbon dioxide, heart‐rate variability and the Shock Index (Convertino et al., 2023; Schiller et al., 2018; Schlotman et al., 2020). More recently, CRM has been successfully implemented using photoplethysmographic waveforms, including a study of 49 healthy adults (25 women), supporting the feasibility of noninvasive and potentially wearable reserve monitoring (Roden et al., 2024).

Translation of these experimental findings into clinical trauma populations has produced more heterogeneous results. In a prospective observational study of 89 patients meeting trauma‐centre activation criteria at a single Level I trauma centre, the CRI implementation demonstrated superior discrimination compared with systolic blood pressure for predicting haemorrhage‐related life‐saving interventions, with a sensitivity of 83%, a negative predictive value of 91% and an area under the receiver‐operating‐characteristic curve (ROC AUC) of 0.83 versus 0.62 for systolic blood pressure (Johnson et al., 2018). In contrast, a prospective prehospital study of 696 adult injured patients transported to a Level I trauma centre, of whom 21% met the study definition of haemorrhagic shock, found a minimum CRI‐trend AUC of 0.65, which was interpreted as inferior to systolic blood pressure (0.74) and Shock Index (0.72) (Latimer et al., 2023). These contrasting findings suggest that, despite consistently strong experimental performance, the clinical utility of CRM requires further prospective validation across diverse trauma populations.

CRM also has practical limitations. The underlying algorithms are proprietary and require high‐quality arterial pressure or photoplethysmographic waveforms. Furthermore, the machine‐learning algorithms are not fully transparent, which may complicate independent validation, external verification and implementation across different monitoring platforms. Overall, CRM currently has the most extensive experimental evidence among the reserve‐monitoring approaches considered in this review and has demonstrated consistent reserve‐ related behaviour in controlled experimental studies, with clinical performance remaining heterogeneous.

### 2.7 Experimental and clinical evaluation of CVRI

The first pilot empirical evaluation of the Cardiovascular Reserve Hypothesis demonstrated that the CVRI varied predictably with changing physiological demand, providing preliminary support for the feasibility of estimating cardiovascular reserve using routinely obtainable physiological variables. These pilot observations established the physiological rationale for subsequent experimental and clinical investigations (Gabbay et al., 2015).

Experimental studies subsequently evaluated CVRI across diverse physiological stress models. During cardiopulmonary exercise testing (CPET), CVRI declined progressively with increasing workload, reached its lowest values at peak exercise and recovered during the recovery phase. Resting CVRI followed an ordinal pattern across exercise‐capacity groups, with the highest values in participants with normal exercise capacity and progressively lower values in those with mildly, moderately and severely reduced exercise capacity (Segel et al., 2017). This association remained independent of age and sex in multivariable analysis. In contrast, peak‐exercise CVRI was similar across exercise‐capacity groups. During the first 2 min of step‐down recovery, CVRI increased, with the magnitude of recovery being greater in participants with better exercise capacity. These findings provide physiological proof‐of‐concept support for the predicted dynamic behaviour of CVRI during exercise and recovery and suggest that resting CVRI may provide information related to exercise capacity, although they do not constitute clinical evaluation of a diagnostic or prognostic application (Segel et al., 2017).

In a controlled swine haemorrhage model, CVRI declined progressively throughout blood loss and appeared to identify impending circulatory deterioration before overt changes in conventional hemodynamic variables, including cardiac output, which remained relatively stable during progressive haemorrhage (Nadler et al., 2017). This study provides preclinical physiological evidence of reserve tracking during progressive blood loss but cannot establish clinical diagnostic accuracy or clinical utility in humans. Together, these experimental findings support the concept that reserve‐based monitoring reflects progressive recruitment and eventual depletion of cardiovascular compensatory mechanisms across diverse forms of physiological stress.

Clinical evaluation of CVRI has been limited to relatively small studies. In a prospective observational cohort of 71 adult trauma casualties at a single Level I trauma centre, 5 patients had massive haemorrhage on arrival, 5 subsequently developed haemorrhage, and 61 had no significant haemorrhage. CVRI was associated with subsequent development of haemorrhage and demonstrated an AUC of 0.82, compared with 0.88 for lactate, while most other conventional variables demonstrated lower AUCs (Shaya et al., 2024). These findings provide preliminary positive clinical evaluation of incremental prognostic information but remain limited by the small, single‐centre observational design.

In a separate analysis of the Israeli National Trauma Registry, CVRI provided prognostic information beyond traditional trauma scoring systems, suggesting potential complementary value for early risk stratification (Gabbay et al., 2019).

CVRI has been evaluated in conditions characterized by increased cardiovascular demand. Studies of heat stress demonstrated significant associations between lower CVRI values and heat intolerance. In the prospective study by Schiffmann et al., involving 95 young men (16 heat‐intolerant and 79 heat‐tolerant), CVRI showed a partial ROC AUC of 93.2% in the high‐sensitivity region (Schiffmann et al., 2021). The earlier conference‐abstract study also reported an association between lower CVRI and heat intolerance (Epstein et al., 2015). These findings extend CVRI evaluation beyond haemorrhage to physiological stress caused by increased cardiovascular demand but remain preliminary.

Conference abstract data have also suggested that serial CVRI measurements may improve mortality prediction in septic shock when combined with admission characteristics (Raphaeli et al., 2022). However, these findings remain preliminary and should be considered hypothesis‐generating until confirmed in peer‐reviewed prospective studies.

Taken together, these studies suggest that CVRI exhibits physiologically consistent behaviour across diverse forms of cardiovascular stress.

### 2.8 Overall evidence

Collectively, available evidence indicates that CRM and CVRI exhibit physiological behaviour consistent with progressive utilization or depletion of cardiovascular reserve, although the strength and type of evidence differ substantially between technologies and study settings. CRM has the most extensive experimental evidence, particularly from controlled hypovolemia models, whereas CVRI has been evaluated across a broader range of physiological and pathophysiological stressors, generally in smaller experimental and clinical studies. Experimental and preclinical findings provide evidence of physiological behaviour, whereas the available clinical studies provide preliminary diagnostic or prognostic associations. The broader range of CVRI applications should not be interpreted as evidence of greater validation, because most studies remain small, observational, experimental or preliminary and independent replication is limited. Although seemingly promising, current evidence remains limited by small clinical cohorts, heterogeneous study designs and limited independent validation. Importantly, neither CRM nor CVRI directly measures all components of the broader physiological construct of cardiovascular reserve; rather, each provides a model‐dependent surrogate estimate derived from selected physiological signals. Neither technology has yet demonstrated that reserve‐guided monitoring improves patient outcomes. Large prospective multicenter studies will be required to establish their incremental clinical value.

### 2.9 Clinical implications and future directions

Conventional physiological monitoring is designed to detect established physiological abnormalities but may fail to identify patients whose compensatory mechanisms are progressively being exhausted. As highlighted in a landmark review of haemorrhagic shock, arterial pressure may remain within the normal range until more than 30% of circulating blood volume has been lost, illustrating the limitations of relying solely on conventional vital signs (Cannon, 2018).

Beyond acute haemorrhage, the reserve‐based framework may also provide a useful perspective for understanding cardiovascular disorders characterized by impaired physiological reserve. Patients with heart failure frequently maintain apparently stable resting hemodynamics despite markedly reduced capacity to augment cardiac output and tolerate additional physiological stress. Likewise, acute coronary syndromes and cardiogenic shock may represent progressive exhaustion of compensatory cardiovascular reserve before overt circulatory collapse develops. Aging and frailty similarly reduce reserve, increasing susceptibility to physiological decompensation despite relatively preserved resting physiology. Although reserve‐monitoring technologies have not yet been systematically validated in these conditions, they represent clinically important targets for future investigation.

Both the CRM and the CVRI were independently developed to estimate remaining cardiovascular reserve rather than current physiological status. Despite fundamentally different methodologies, machine‐learning analysis of arterial waveform morphology for CRM and integration of routinely measured physiological variables for CVRI, both demonstrate a broadly consistent pattern of progressive decline during increasing physiological stress. Experimental studies suggest that reserve depletion precedes overt hemodynamic decompensation, supporting the concept that reserve‐based monitoring may identify circulatory deterioration earlier than conventional monitoring (Convertino et al., 2023; Epstein et al., 2015; Nadler et al., 2017; Roden et al., 2024; Schiffmann et al., 2021; Schlotman et al., 2020; Segel et al., 2017; Shaya et al., 2024).

The convergence of findings across diverse experimental and early clinical settings provides preliminary support for the hypothesis that cardiovascular reserve may represent a measurable physiological construct reflecting the balance between compensatory capacity and physiological demand. CRM has accumulated the strongest experimental evidence, particularly in controlled hypovolemia models, whereas CVRI has demonstrated physiologically consistent behaviour across exercise, haemorrhage and heat stress. However, whether these approaches quantify the same physiological construct, complementary dimensions of cardiovascular compensation or distinct aspects of physiological reserve remains unknown.

If future studies confirm these observations, reserve‐based monitoring could complement conventional physiological assessment by identifying patients approaching circulatory decompensation before overt hemodynamic instability becomes clinically apparent.

### 2.10 Limitations and future research

No accepted gold‐standard measure of cardiovascular reserve currently exists, making direct validation inherently challenging. The available evidence is heterogeneous and consists largely of experimental studies, pilot investigations and relatively small observational cohorts, with limited external validation and few direct comparisons between reserve‐monitoring technologies. The studies also differ substantially in experimental model, physiological stressor, population, outcome definition and comparator measures, limiting direct quantitative comparison across studies.

Perhaps the greatest limitation is the absence of outcome studies demonstrating that reserve‐guided monitoring improves clinical decision‐making or patient‐centred outcomes. In addition, optimal decision thresholds, measurement reliability and generalizability remain incompletely defined, and whether CRM and CVRI quantify the same underlying physiological construct remains unknown.

Although reserve‐monitoring technologies have demonstrated encouraging findings across multiple physiological stress models, their clinical role remains investigational. Future research should establish whether cardiovascular reserve constitutes a robust, measurable and clinically actionable physiological construct across diverse disease states, and whether CRM and CVRI quantify a common dimension of cardiovascular reserve or capture complementary aspects of cardiovascular compensation.

Because cardiovascular reserve reflects the dynamic balance between physiological capacity and physiological demand, its validity should be examined across conditions that challenge different components of cardiovascular compensation. Accordingly, future validation studies should evaluate reserve‐monitoring technologies across a broad spectrum of physiological and pathological stress models, including:

*Low‐output states*: haemorrhage, hypovolemia, dehydration and cardiogenic shock.

*Pathological high‐output states*: sepsis, anaphylaxis and advanced liver failure.

*Physiological reserve‐utilization models*: exercise and orthostatic stress.

*High‐demand physiological stress models*: heat stress, hyperthermia and high‐altitude exposure.

*Oxygen delivery–utilization mismatch states*: severe anaemia, hypoxaemia, acute respiratory failure and carbon monoxide poisoning.

*Impaired autonomic compensation*: autonomic failure, spinal cord injury and β‐adrenergic blockade.

*Chronic reserve‐depletion states*: heart failure, chronic pulmonary disease, frailty and aging.

*Transitional physiological states*: major surgery, pregnancy and the immediate postpartum period.

Evaluation across these complementary models will help establish the physiological generalizability of reserve monitoring, identify the clinical settings in which reserve estimation provides the greatest incremental value beyond conventional monitoring and determine whether CRM and CVRI measure the same physiological construct or provide complementary information regarding cardiovascular reserve.

Ultimately, widespread clinical adoption of reserve‐monitoring technologies will depend not only on demonstrating accurate estimation of cardiovascular reserve, but also on proving that reserve‐guided management improves clinical outcomes beyond existing monitoring strategies.

## 3 CONCLUSIONS

Reserve‐based monitoring represents a conceptual shift from assessment of current physiological status toward estimation of remaining compensatory capacity. Among currently available technologies, CRM has the strongest experimental evidence base, whereas CVRI offers a physiologically grounded and operationally simple alternative that has demonstrated preliminary physiological and clinical findings across exercise, heat stress, haemorrhage and trauma. Although current findings support the physiological plausibility of reserve estimation, both approaches remain investigational owing to limited independent validation and the absence of outcome‐based clinical trials. The available evidence should therefore be interpreted as supporting physiological plausibility and feasibility rather than established clinical utility.

Whether compensatory reserve represents a single measurable physiological construct or a family of related physiological phenomena remains an important question for future investigations. Nevertheless, the convergence of multiple independently developed reserve‐monitoring methodologies supports continued investigation of cardiovascular reserve as a measurable and potentially clinically relevant physiological construct.

## CONFLICT OF INTEREST STATEMENT

Uri Gabbay is the inventor of the Cardiovascular Reserve Index (CVRI), holds intellectual property related to the technology and has participated in efforts to commercialize the technology through CardioScale. Several studies discussed in this review involved investigators associated with the development or evaluation of CVRI. To mitigate potential bias, competing reserve‐monitoring approaches, methodological limitations, conflicting findings and areas of uncertainty have been explicitly discussed. The remaining authors declare no conflicts of interest.

## Supporting information

Supporting file 1.

## Data Availability

No new datasets were generated or analysed for this narrative review. All information discussed is derived from the cited published literature.
