# Supplementary material for: Cardiovascular reserve as an integrative physiological construct: Current evidence, emerging technologies and future directions
Source: Clin Physiol Funct Imaging. 2026 Oct 4;46(6):e70094. doi: 10.1111/cpf.70094 (PMC13635602; doi:10.1111/cpf.70094)
Supplement: Supplementary file 1 [file CPF-46-0-s001.docx]

**Appendix 1**

**Physiological Basis and Derivation of the Cardiovascular Reserve Index (CVRI)**

**A1. Cardiovascular Reserve Hypothesis**

The Cardiovascular Reserve Hypothesis provides the conceptual framework from which the Cardiovascular Reserve Index (CVRI) was developed, proposing that progressive depletion of cardiovascular reserve precedes overt physiological instability and that shock represents the endpoint of reserve exhaustion and loss of compensatory capacity [Gabbay et al. 2014].

The derivation presented below summarizes the conceptual physiological rationale underlying CVRI and is intended to provide physiological context rather than formal mathematical proof.

**A1.1 Origins of the Cardiovascular Reserve Concept**

The conceptual origins of CVRI derive from investigations of cardiovascular control systems and physiological reserve using systems-control theory. Dvir et al. described a relationship between low-frequency heart-rate variability and cardiovascular open-loop gain (OLG), a theoretical parameter representing the capacity of the cardiovascular control system to augment performance in response to physiological stress. OLG was subsequently proposed as a theoretical construct that may reflect aspects of cardiovascular reserve [Dvir et al, 2013].

**A1.2 Open-Loop Gain as a Reserve Model**

Cardiovascular open-loop gain (OLG) was proposed as a theoretical representation of cardiovascular reserve and modeled as proportional to:

*OLG ∝ SV × SVR × BRS*	[Dvir et al, 2013].

*where SV denotes stroke volume, SVR denotes systemic vascular resistance, BRS denotes baroreflex sensitivity*.

To account for body size, OLG was normalized (OLGN) by body surface area (BSA):

*OLGN ∝ (SV × SVR × BRS)/BSA*

Because stroke volume, systemic vascular resistance, and baroreflex sensitivity are not routinely measurable in clinical practice, the CVRI formulation substitutes clinically accessible surrogate variables while preserving the underlying physiological framework.

**A1.3 Derivation of CVRI**

*According to standard hemodynamic relationships*

$$SV\times SVR=\frac{80\times VPG}{HR}$$

*where VPG is the vascular pressure gradient, HR is heart rate.*

Respiratory rate was incorporated as a pragmatic surrogate of cardiorespiratory and autonomic responses to physiological stress. Its inclusion was based primarily on physiological plausibility and empirical observations during model development rather than direct evidence that respiratory rate independently quantifies cardiovascular reserve.

$$SV\times SVR\times BRS=\frac{80\times VPG}{HR\times RR}$$

*A scaling factor of 4 was introduced empirically to normalize CVRI values around unity.*

*The resulting formulation is:*

$$CVRI=\frac{20\times VPG}{HR\times RR\times BSA}$$

VPG is defined as the systemic vascular pressure gradient:

VPG = MAP – RAP

where RAP denotes right atrial pressure.

Under normal physiological conditions, this is approximately equivalent to:

VPG = MAP – CVP

where CVP denotes central venous pressure.

For noninvasive implementation, the systemic pressure gradient was approximated using a fixed proportion of MAP as described in the original derivation [Gabbay et al. 2015].

VPG ≈ 0.9 × MAP

This approximation was adopted to permit practical noninvasive implementation and should be regarded as a simplifying assumption rather than a direct measurement of central hemodynamics. It is not intended to estimate central venous pressure directly.

**A1.4 Physiological Interpretation**

CVRI increases when cardiovascular capacity is relatively preserved and decreases as compensatory mechanisms become progressively utilized. Accordingly, higher values are interpreted as greater available reserve, whereas lower values suggest greater reserve utilization. CVRI should be regarded as a surrogate estimate of cardiovascular reserve rather than a direct measurement of any individual physiological parameter.

**A1.5 Practical Calculation Considerations**

CVRI was designed to permit calculation from routinely available physiological variables. Although simplifications are required for noninvasive implementation, the objective is not precise estimation of individual hemodynamic variables, but preservation of information related to overall compensatory reserve.

Importantly, the derivation presented here should be regarded as a conceptual physiological model intended to generate testable hypotheses rather than a definitive mechanistic proof of cardiovascular reserve.

**References**

Dvir, H., Bobrovsky, B.Z. & Gabbay, U. (2013) A novel heart rate control model provides insights linking LF‐HRV behavior to the open‐loop gain. International Journal of Cardiology, 168(1), 287–293.
